# A novel mutation in the matrix metallopeptidase 2 coding gene associated with intrafamilial variability of multicentric osteolysis, nodulosis, and arthropathy

**DOI:** 10.1002/mgg3.802

**Published:** 2019-07-03

**Authors:** Liisa Kröger, Tuija Löppönen, Leena Ala‐Kokko, Heikki Kröger, Hanna‐Mari Jauhonen, Kaisa Lehti, Jarmo Jääskeläinen

**Affiliations:** ^1^ Department of Pediatrics Kuopio University Hospital Kuopio Finland; ^2^ Department of Child Neurology Kuopio University Hospital Kuopio Finland; ^3^ University of Eastern Finland Kuopio Finland; ^4^ Connective Tissue Gene Tests Allentown Pennsylvania; ^5^ Department of Orthopedic Surgery Kuopio University Hospital Kuopio Finland; ^6^ Finnish Medicines Agency Kuopio Finland; ^7^ University of Helsinki and Helsinki University Hospital, Genome‐Scale Research Program Helsinki Finland

**Keywords:** arthropathy, joint contractures, matrix metallopeptidases, MONA, Osteolysis

## Abstract

**Background:**

MONA, which stands for a spectrum of Multicentric Osteolysis, subcutaneous Nodulosis, and Athropathia, is an ultra rare autosomal recessive disorder caused by mutations in the matrix metallopeptidase 2 (*MMP2*) gene. To date only 44 individuals, carrying 22 different mutations have been reported. Here we report on two brothers with identical homozygous *MMP2* gene mutations, but with clearly different phenotypes.

**Methods:**

Genomic DNA was isolated from the affected brothers and the parents. An iliac crest bone biopsy was taken from the younger patient (index case). The level of matrix metallopeptidase 2 enzyme (MMP2) in serum and synovial fluid of the younger patient was analyzed using gelatin zymography.

**Results:**

The DNA analysis revealed a homozygous c.1188C>A transversion on exon 8 of the gene. The affected brothers had the same homozygous variant and the parents were heterozygous to this variant. This variant has been reported as a compound heterozygous mutation on one individual resulting in scleroderma like skin thickening. Bone histomorphometry indicated increased trabecular bone remodeling and turnover. The zymography revealed that the level of MMP2 was completely nonmeasurable in the serum and only a minor gelatinolytic protein band of about similar molecular weight as MMP2 was found in the synovial fluid.

**Conclusions:**

Both the age at the onset and the phenotypic severity of the syndrome in these two brothers were different despite identical genotypes. The younger patients had corneal opacities leading to deteriorating visual acuity. For the first time in this disease, opacities were successfully treated with corneal transplantations.

## 1 INTRODUCTION

MONA—Multicentric Osteolysis, Nodulosis, and Arthropathy (OMIM no. 259600)––is a rare autosomal recessive disorder (Bhavani et al., 2015; Zankl et al., 2007). It is characterized by osteoporosis and osteolysis particularly in the carpal and tarsal bones, progressive joint contractures, and subcutaneous nodules. Such additional features as gingival hypertrophy, pigmented skin lesions, coarse face, corneal opacities, and cardiac defects have been reported (Bhavani, Shah, Shukla, & Girisha, 2016; Tuysuz et al., 2009). In most children the onset of symptoms is between the ages of 6 months and 6 years (Azzollini et al., 2014; Bhavani et al., 2016). The differential diagnoses include juvenile idiopathic arthritis (joint contractures) and mucopolysaccharidoses (dysmorphic features and radiological findings) (Bhavani et al., 2016; Castberg et al., 2013; Zankl et al., 2007).

Multicentric Osteolysis, Nodulosis, and Arthropathy is caused by inactivating mutations in the matrix metallopeptidase2 (*MMP2*) gene. To date, 22 different *MMP2* mutations associated with MONA have been reported (Bhavani et al., 2016, 2015).

We report two siblings carrying an identical homozygous inactivating *MMP2* mutation both diagnosed with MONA but with clear phenotypic differences. We also report for the first time bone histomorphometry findings in MONA. One of the siblings was treated with corneal transplantation, for the first time in this condition.

A two‐year‐old boy (Patient 1), the youngest of three children, presented first with subcutaneous nodules in the gluteal area, followed by limping and contractures of the metacarpal joints. He had a history of a conservatively treated lower leg fracture 6 months earlier.

**Figure 1:**
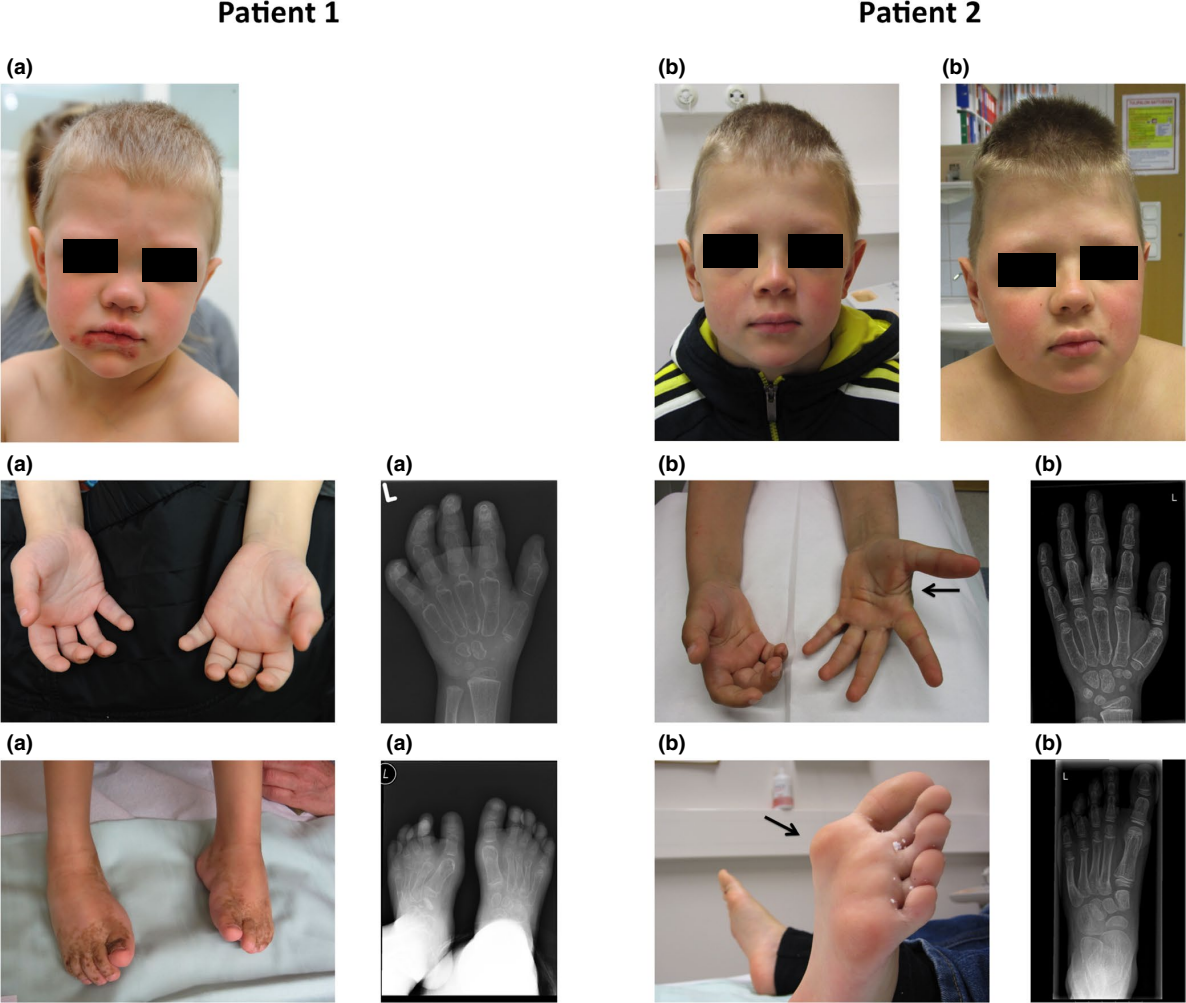
Patient 1 (a) at the age of 3 years and his older brother (Patient 2) (b) at the ages of 8 and 11 years both showing minor facial dysmorphic features: bulbous nose and frontal bossing. The younger patient (a) had joint contractures in both hands and feet. The older one (b) suffered from painful subcutaneous nodules in the plantar area and tendon restrictions in the hands. Radiographs demonstrate bone loss, wide metacarpals, and metatarsal bones

Musculoskeletal examination revealed flexion contractures in the proximal and distal interphalangeal joints in the hands and feet as well as in the wrists and ankle joints. The skin of the soles and palms was thickened. Semiflexion in the knees and elbows was registered. He also had periorbital edema and coarse facial features (Figure 1).

Radiography of the hands and feet demonstrated widening of the tubular bones and generalized osteopenia (Figure 1). Ophthalmological examination showed bilateral corneal opacities and visual impairment. An audiogram, neurodevelopmental examination, spinal X‐ray and MRI, head MRI, and cardiac ultrasound were normal. MONA was confirmed by genetic analysis. By the age of 8 years he had suffered metatarsal and femoral fractures, both following minor traumas. He has scoliosis and is of relatively short stature (height has varied from −0.1 to −1.1 SDs, BMI from −1.3 to −2.2 SDS, and head circumference from −1.3 to 0.0 SDS). He developed gingival hypertrophy and pigmented skin lesions. During the follow‐up, joint contractures in wrists, elbows, knees, and ankles had progressed only slightly due to intensive physiotherapy and occupational therapy and there has even been an improvement in the mobility of the metacarpal and finger joints.

During follow‐up, the opacities had progressed in both eyes and his right eye was operated on at the age of 4 years. The operation improved his vision from severely impaired (hand movement to light perception) to 0.08 (Snellen visual acuity). The pannus progressed in his left eye and was operated on later. His visual acuity at the age of 8 years was 0.4 in the right eye both for distance and close‐up. At the moment, his left eye is amblyopic but he is able to attend school with the help of visual rehabilitation devices and with a personal assistant.

This patient´s two older brothers, then aged 6 and 8 years, underwent clinical examination shortly after the diagnosis of MONA syndrome was made. The parents were not willing to give permission for predictive genetic testing of these children. However, at the age of 8 years, the middle brother suffered a metacarpal fracture following a minor trauma. Hand X‐rays showed widening and osteoporosis of the metacarpal bones, raising the suspicion of MONA. Furthermore, foot X‐rays showed osteolysis and the suspicion of a healed fracture in the third metatarsal. After thorough discussion and consideration his parents agreed to DNA testing for this child and the same mutation was found. On follow‐up he suffered from an increasing amount of subcutaneous nodules in his palms and soles and had developed palmar tendon restrictions. He has also been treated for forearm and humeral fractures. Some facial features such as hypertelorism and frontal bossing have become more prominent (Figure 1). However the clinical findings are significantly milder than in his younger brother. He has been growing relatively well: his height −1.5 SDS, BMI +2.4 SDS, and head circumference 0.0 SDS. Bone mineral density (BMD) values at the lumbar spine and femoral neck were at the lower end of the age reference.

**Figure 2:**
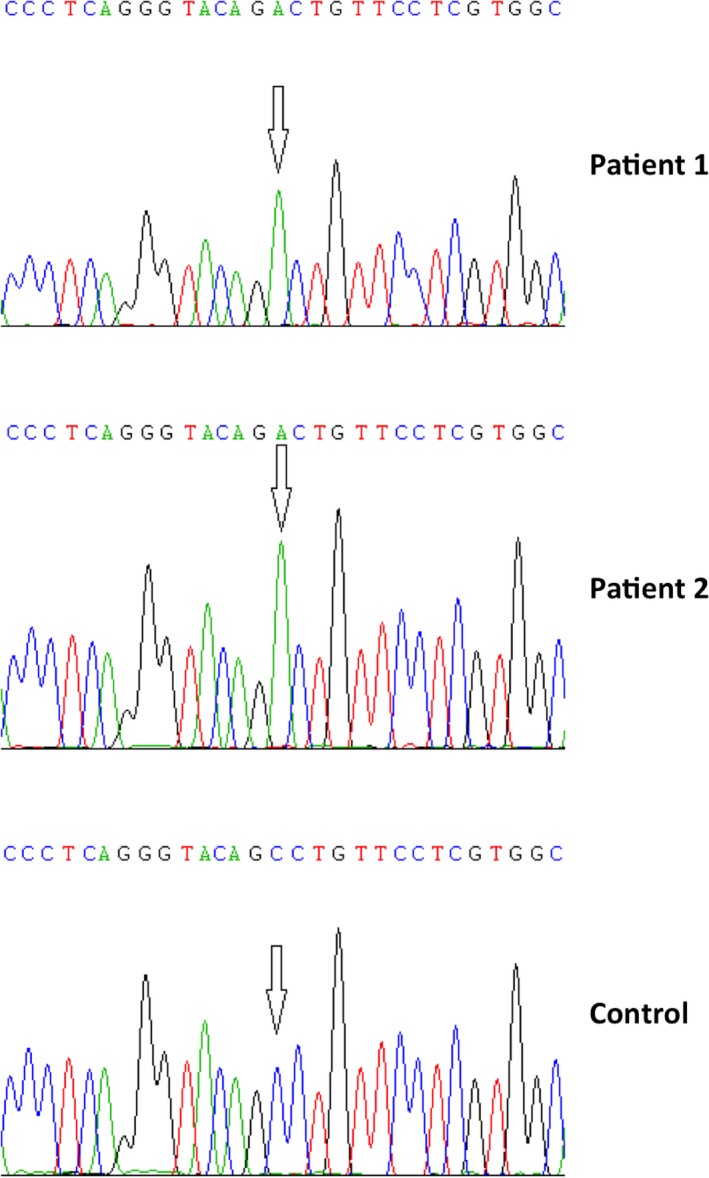
Chromatograms show a homozygous c.1188C>A mutation in the *MMP2* gene

Genomic DNA was isolated from the affected brothers and their parents and analyzed for sequence variations in the *MMP2* gene. The DNA analysis revealed a homozygous c.1188C>A transversion in exon 8 of the gene (NM_004530.5) resulting in S396R substitution (Figure 2). Both affected brothers had the same homozygous variant and the parents were heterozygous for the variant. This variant has been recently reported as a compound heterozygous mutation in one individual with the MONA phenotype (Bader‐Meunier et al., 2016). This change is also listed in the genomAD database with an A allele frequency of approximately 0.16% in 25,792 Finnish chromosomes. Comparison of *MMP2* amino acid sequences derived from numerous species show that Ser396 is well conserved (54/54 species). Ser396 is located in the catalytic domain of the protein.

**Figure 3:**
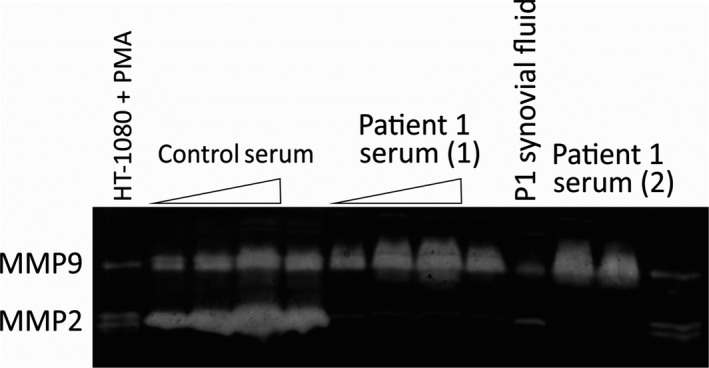
The level of matrix metallopeptidase 2 (MMP2) in aliquots of serum and synovial fluid from Patient 1 (index case) was analyzed using zymography. The level of MMP2 was very low in synovial fluid and was completely nonmeasurable in the serum. Normal human serum with abundant MMP2 activity was used as a control (serum aliquots of 0.5, 1, and 2 μl were used as indicated by the triangle; two different serum samples for the MONA index case were analyzed as indicated). Conditioned medium from phorphol ester treated (PMA; 4 nm) HT‐1080 fibrosarcoma cells served as a control for MMP9 and MMP2 (72 kDa pro‐form and 62 kDa active enzyme were separated by protein migration in electrophoresis prior to in‐gel activation and gelatinolysis (Lohi et al., 1996))

The level of matrix metallopeptidase 2 enzyme in serum and synovial fluid of Patient 1 was analyzed using gelatin zymography as described (Lohi, Lehti, Westermarck, Kähäri, & Keski‐Oja, 1996). Only a minor gelatinolytic protein band of about similar molecular weight as MMP2 was found in the synovial fluid and the level of MMP2 was completely nonmeasurable in the serum (Figure 3).

**Figure 4:**
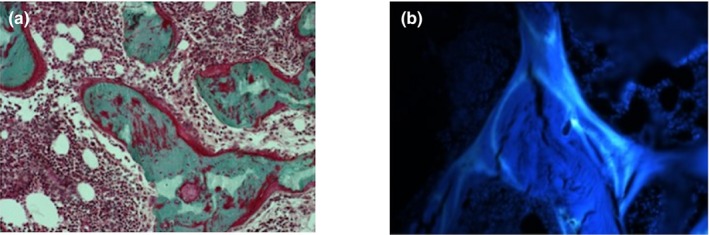
Bone histomorphometry images from Patient 1 at the age of 4 years. Undecalcified sections: (a) Light microscopy (200X) showing mineralized (green) trabeculae, covered by osteoid (red). Active osteoblasts and osteoclasts can be seen on bone surfaces. Undecalcified sections and modified Masson‐Goldner staining. (b) Fluorescence microscopy (200x) showing tetracycline labels indicating bone formation and mineralization

An iliac crest bone biopsy was taken from the index case (Patient 1) at the age of 4. Our bone histomorphometry protocol, analysis, and used pediatric reference data have been reported previously (Glorieux et al., 2000; Mäyränpää, Tamminen, Kröger, & Mäkitie, 2011). Trabecular bone volume (BV/TV) was normal (20.8%, [normal mean ± *SD*, 17.7 ± 2.6]). Osteoid volume (OV/BV 11.7% [4.0 ± 1.2]) and osteoid thickness (O.Th, 10.0 µm [5.8 ± 1.4]) were elevated and there was a large number of active osteoblasts (Ob.S/BS, 12.2%) (8.5 ± 4.1). Osteoid surface (OS/BS) was 39.2% (34.0 ± 6.7). Erosion surface (ES/BS) 9.3% (14.8 ± 4.4) and the amount of active osteoclasts was high (Oc.S/BS, 10.0% [1.1 ± 0.8]). Tetracycline double labeling in vivo was performed prior to biopsy. UV‐microscopy showed increased tetracycline uptake: mineralization surface (MS/BS, 29.7% [12.5 ± 4.5]), mineralization apposition rate (MAR, 1.41 µm/day [1.0 ± 0.2]), and activation frequency (Ac.f, 4.5/year [1.4 ± 0.5]). Histomorphometry findings suggested increased trabecular bone remodeling and turnover in this patient. No signs of mineralization defects were detected (Figure 4).

Here we report two brothers diagnosed with MONA, which is an ultra‐rare disorder; only 44 cases from 27 families carrying 22 different mutations have been reported previously (Bhavani et al., 2016). Most affected individuals have carried homozygous mutations and are children of consanguineous parents. In our case, there is no known consanguinity.

Most children with MONA appear normal at birth and the onset of symptoms has varied from 6 months to 11 years (Bhavani et al., 2016). The phenotype of our younger patient was more severe and he was diagnosed much younger than his older brother. This confirms the observation that the clinical phenotype and the age at the onset of symptoms can vary widely in the presence of identical mutation (Bhavani et al., 2016, 2015).

In our younger patient, ophthalmologic examination revealed broad central opacity in both eyes. Corneal opacities are occasionally observed in MONA patients but vision is normally preserved. In our patient the corneal lesions progressed and led to deteriorating visual acuity. As the progression in this patient was unusual, other etiologies cannot be totally excluded. Corneal transplantation was successfully performed with encouraging results.

Matrix metallopeptidases are extracellular zinc‐dependent peptidases with a role in the development and repair of the skeleton. MMP2 can degrade various extracellular matrix components of interstitial connective tissues and basement membranes such as collagens, fibronectin, laminin, and aggrecan as well as extracellular soluble proteins including inflammatory mediators. The denaturation of collagen is important for collagen turnover. As bone formation and resorption are tightly coupled events, incomplete collagen degradation can cause osteoblast dysfunction and an imbalance in bone turnover. *MMP2*‐null mice have presented with decreased bone mineralization, articular cartilage destruction, joint erosion, and defects of osteoblast and osteoclast growth (Mosig et al., 2007). However, the findings in this study are partly opposite to this, since Patient 1 showed an increased bone turnover with profuse osteoblast and osteoclast surfaces and no signs of decreased mineralization. Bone biopsy was obtained from the iliac crest, which is the standard location for quantitative bone histomorphometry. It does not necessarily represent bone remodeling status in the affected bones. Similarly, central bone density does not represent peripheral bone status.

MMP2 proteolytic activity measured using gelatin zymography was completely unmeasurable in serum of our index patient, but a minor gelatinolytic protein band of similar mobility to MMP2 in the nonreducing gel electrophoresis for zymography was present in the synovial fluid. Future investigation will be needed to assess, whether this protein band represents (unlikely) residual MMP2 activity, or another protease such as a cathepsin with gelatinolytic activity in the zymography.

Because the clinical findings in the early course of the disease mimic severe juvenile idiopathic arthritis many patients diagnosed as suffering from MONA spectrum disorders have previously been treated with anti‐inflammatory drugs, systemic corticosteroids, and methotrexate, naturally without long‐term beneficial effects (Castberg et al., 2013). As there is wide clinical variation in MONA syndrome, it is important to consider this hereditary disorder when examining patients with such joint swellings or contractures, which are not typical of inflammatory arthritis, patients with subcutaneous nodules or even with such special features as thickening of the skin (Bader‐Meunier et al., 2016).

As there are several reports of successful bisphosphonates therapy in such bone diseases as osteogenesis imperfecta, the effect of cyclic intravenous pamidronate has been evaluated in children with MONA (Al‐Mayof, Madi, & Bin‐Abbas, 2006). The treatment has been well‐tolerated but bone mineral density has not improved significantly and the osteolytic changes have not improved (Al‐Mayof et al., 2006). However, bisphosphonates may reduce skeletal pain and may thus be considered together with NSAIDs and physical therapy (Pichler et al., 2016).

In conclusion, MONA is an ultra‐rare severe progressive disease and limited data are available on its natural course (Bhavani et al., 2016, 2015). The variation in phenotypes may be wide even with identical genotypes. There is no specific alleviating treatment but multidisciplinary treatment may improve patients’ quality of life. Further improvements in understanding bone metabolism in this condition may offer new treatment options.

## CONFLICT OF INTEREST

All authors declare no conflict of interest.
